# Earliness *per se*×temperature interaction: consequences on leaf, spikelet, and floret development in wheat

**DOI:** 10.1093/jxb/erz568

**Published:** 2019-12-26

**Authors:** Paula Prieto, Helga Ochagavía, Simon Griffiths, Gustavo A Slafer

**Affiliations:** 1 Department of Crop and Forest Sciences, University of Lleida - AGROTECNIO Center, Lleida, Spain; 2 John Innes Centre, Norwich Research Park, Norwich, UK; 3 ICREA, Catalonian Institution for Research and Advanced Studies, Spain; 4 University of Nottingham, UK

**Keywords:** *Earliness* per se (Eps), floret development, phyllochron, plastochron, spikelet number, *Triticum aestivum*

## Abstract

Wheat adaptation can be fine-tuned by *earliness per se* (*Eps*) genes. Although the effects of *Eps* genes are often assumed to act independently of the environment, previous studies have shown that they exhibit temperature sensitivity. The number of leaves and phyllochron are considered determinants of flowering time and the numerical components of yield include spikelets per spike and fertile floret number within spikelets. We studied the dynamics of leaf, spikelet, and floret development in near isogenic lines with either late or early alleles of *Eps*-*D1* under seven temperature regimes. Leaf appearance dynamics were modulated by temperature, and *Eps* alleles had a greater effect on the period from flag leaf to heading than phyllochron. In addition, the effects of the *Eps* alleles on spikelets per spike were minor, and more related to spikelet plastochron than the duration of the early reproductive phase. However, fertile floret number was affected by the interaction between *Eps* alleles and temperature. So, at 9 °C, *Eps*-early alleles had more fertile florets than *Eps*-late alleles, at intermediate temperatures there was no significant difference, and at 18 °C (the highest temperature) the effect was reversed, with lines carrying the late allele producing more fertile florets. These effects were mediated through changes in floret survival; there were no clear effects on the maximum number of floret primordia.

## Teaser

Temperature can reverse the allelic effect of *Eps* genes. At low temperatures, early alleles of *Eps-D1* are associated with increased fertile floret number but at higher temperatures with less.

## Introduction

Adaptation to a wide range of environmental conditions is critical for wheat which is grown in most arable lands of the world and all of which are experiencing further change in growing conditions as a consequence of climate change (Asseng *et al.*, 2015; Hernandez-Ochoa *et al.*, 2019; Leng and Hall, 2019). Major genetic factors controlling adaptability involve photoperiod and vernalization sensitivity (*Ppd* and *Vrn*) and earliness *per se* (*Eps*) genes. The latter, normally with smaller effects, are quite relevant in regions where the crop has been already reasonably well adapted as they allow for fine-tuning adaptation (Slafer, 2012; Gomez *et al.*, 2014; Zikhali and Griffiths, 2015). Early flowering due to the action of early alleles of *Eps* genes was reported in *Triticum aestivum* by Zikhali *et al.* (2015) due to a deletion of the chromosomal region including *ELF3* corresponding to the earliness *per se* locus *Eps*-*D1*.

The *Eps-A*^*m*^*1* from cultivated *Triticum monococcum*, colinear to *Eps*-*D1* (Alvarez *et al.*, 2016, and references therein), was reported not only to have an unusually large effect on plant development but also to interact with temperature (Bullrich *et al.*, 2002; Appendino and Slafer, 2003; Lewis *et al.*, 2008). Moreover, the *Eps-3A*^*m*^ loci were also reported to affect development, but the magnitude of the effect depended on the growth temperature (Gawroński *et al.*, 2014).

Thus, even though an *Eps* gene is expected to produce an effect on developmental traits independent of the environment (and that is why it was named ‘*per se*’; Slafer, 1996), at least in *T. monococcum* (in which these genes have a rather large effect on phenology) this assumption was proven wrong. Evidence for *Eps*×temperature interaction in hexaploid wheat, in which the genetic effects are ‘minor’, had not been reported until now. We recently grew near isogenic lines (NILs) differing in an *Eps-D1* allele that had been shown to affect time to heading differently depending on the field conditions (Zikhali *et al.*, 2014; Ochagavía *et al.*, 2018*a*) under a range of thermal conditions (Ochagavía *et al.*, 2018*c*). When quantifying *Eps*×temperature interaction in phenology, we showed that these *Eps* genes would probably be temperature sensitivity genes rather than *per se* (Ochagavía *et al.*, 2018*c*). In that work, we studied the effects on time to heading, in addition to the effects on the duration of component phases (from the beginning of the experiment to terminal spikelet and from then to heading). This is relevant because it is during these phases that the initiation of primordia destined to become leaves, spikelets, and florets takes place.

An alternative way to study genetic and environmental effects on time to heading is through analysing the leaf appearance rate (whose reciprocal is the phyllochron, i.e. the interval between the appearance of two successive leaves) and final leaf number (FLN), complemented with the time from flag leaf appearance to heading. In turn, FLN depends on the duration of the vegetative phase to floral initiation and the rate of leaf primordia initiation (whose reciprocal is the leaf plastochron, i.e. the interval between the initiation of two successive leaf primordia). From floral initiation onwards, reproductive structures develop in the apex; first spikelet and then floret primordia (within spikelets) are differentiated. Determining the effects of *Eps* genes on the rates of leaf, spikelet, and floret initiation could show whether or not the effects of these genes on the duration of particular phases would translate into changes in the number of primordia. Knowing these effects could indicate whether fine-tuning adaptation with these genes would bring about compensations in yield potential as (i) leaf primordia and the dynamics of leaf appearance are the basis for the later determination of leaf area and crop growth through radiation interception and (ii) spikelet/floret primordia are the determinants of spike fertility, both sources and sinks relevant to determination of yield (Reynolds *et al.*, 2012; Slafer *et al.*, 2014).

Under field conditions, we found that some *Eps* genes affect these dynamics, but also hypothesized that temperature played a role in modulating the effects of these genes on primordia initiation and final number of organs when comparing across seasons or with studies conducted elsewhere (Ochagavía *et al.*, 2018*a*; Prieto *et al.*, 2018*b*). To the best of our knowledge, no previous studies have determined the effects of *Eps* genes under contrasting temperatures on the dynamics of leaf initiation (and consequently on final leaf number), leaf appearance (determining, together with FLN, time to flag leaf appearance), spikelet initiation (and consequently on the number of spikelets per spike), and of floret generation/degeneration (and consequently on the number of fertile florets).

The main aim of this work was to analyse the effect of the *Eps* genes across a wide range of temperatures on leaf, spikelet, and floret developmental patterns responsible for the number of leaves, spikelets, and fertile florets at anthesis. For this purpose, we grew fully vernalized plants of NILs for *Eps* under contrasting temperatures and a long photoperiod.

## Materials and methods

### General description

Different experiments were carried out under controlled conditions in order to test the effect of *Eps* genes at a wide range of temperatures. Experiments were carried out in growth chambers at the University of Lleida (UdL; Lleida, Spain) and the John Innes Centre (JIC; Norwich, UK). Radiation was 110 μmol m^−2^ s^−1^ of photosynthetically active radiation at the plant level in the UdL, and ~300 μmol m^−2^ s^−1^ in the JIC.

We first vernalized the seedlings (at 4 °C for 49 d). After vernalization, seedlings were transferred to growth chambers and subjected to their corresponding temperature treatments; which was the onset of the experiments. Plants were fertilized and irrigated so that there were no nutrient or water limitations to growth. In all cases, photoperiod was long (18 h).

### Treatments

Treatments consisted of the factorial combination of *Eps* NILs and temperatures. The *Eps* NILs selected resulting from the cross of two double haploid lines derived from the cross Spark×Rialto (SR9 and SR23, both carrying the *Eps*-early allele of Spark in chromosome 1DL) were back-crossed with Rialto (which carries the *Eps*-late allele in 1DL) as the recurrent parent, as described in Zikhali *et al.* (2014). As the two pairs of NILs (derived from SR9 and SR23, carrying both the *Eps*-early and late alleles) had exactly the same responses to temperature (see Ochagavía *et al.*, 2018*c*), to quantify the effects we averaged the results of the two *Eps-*early and the two *Eps-*late NILs.

The temperatures tested at the UdL chambers were 6, 9, 15, 21, and 24 °C, and those imposed at the JIC chambers were 12 °C and 18 °C.

Within each chamber (temperature regime) we arranged the NILs in a completely randomized design with three replicates. Each of these replicates consisted of ~18 (UdL) or 21 (JIC) pots per genotype (a total of 220 and 252 pots per temperature regime in the UdL and JIC, respectively).

### Measurements and analysis

Three plants per replicate of each genotype (in total nine plants per genotype) were labelled at the beginning of the experiments. For all plants, we determined (i) heading (DC59) and anthesis (DC65) following the decimal code developed by Zadoks *et al.* (1974); and (ii) leaf appearance dynamics recording frequently, from the onset of the experiment onwards, the number of leaves that had appeared on the main shoot, following the scale of Haun (1973). The actual frequency of leaf number determinations depended on the temperature, from one (lowest temperature) to four (high temperatures) times per week. Leaf appearance dynamics presented bilinear trends with the break-point at around the appearance of the seventh leaf in most temperature treatments (except at 6 °C and 9 °C). For this reason, two different phyllochrons were calculated for each combination of genotype and temperature as the reciprocal of the first slope (phyllochron of early leaves) and of the second slope (phyllochron of late leaves) of the bilinear regressions (Ochagavía *et al.*, 2018*a*). However, to account for overall effects of treatments, we calculated a weighted average phyllochron.

In order to determine spikelet and floret dynamics, one plant per replicate of each genotype (i.e. three plants per genotype, and six plants for each *Eps* allele) was randomly harvested frequently. The actual frequency depended on the speed of development exhibited by the plants, in turn depending on the temperature regimes.

Plants were dissected to determine the apex stage of development following the scale of Waddington *et al.* (1983) as well as to count the number of primordia in the apex allowing the determination of primordia initiation dynamics. For this purpose, the cumulated number of primordia was related to time since floral initiation and the data were fitted with bilinear regressions. From the bilinear regression, plastochrons (leaf and spikelet plastochron) were calculated as the reciprocal of the first and second slope, respectively (Ochagavía *et al.*, 2018*a*).

**Fig. 1.**
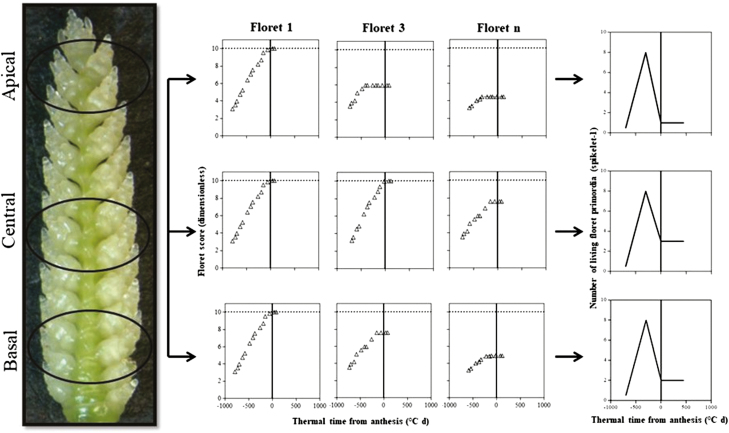
Illustration of the spikelet positions, apical (A–D), central (E–H), and basal (I–L), in which floret development was determined from frequent samplings. Floret score of each floret primordium using the scale of Waddington *et al.*, 1983, and the number of living floret primordia were plotted against thermal time from anthesis (therefore, on the *x*-axis, zero represents thermal time of anthesis, and previous developmental phases took place at negative values of thermal time on this scale) for the *Eps*-early and *Eps*-late NILs in each of the growing conditions. (This figure is available in colour at *JXB* online.)

Floret development dynamics were followed in basal (the fifth position counting from the base of the spike), central (exactly the central spikelet in each spike, when the total number of spikelets was even, we selected the lowest of the two central spikelets), and apical (that immediately below the terminal spikelet) spikelets (Fig. 1), as previously described in detail in Prieto et al., 2018*a*, *b*). The number of floret primordia was counted and the Waddington scale (Waddington *et al.*, 1983) was used to determine the stage of development of each primordium analysed [Floret 1, 2, 3,..*n*; counted from the most proximal (F1) to the most distal (F*n*) with respect to the rachis] (Fig. 1). At anthesis, at least three plants per genotype and replicate (in total under each temperature regime nine plants per genotype and 18 plants per *Eps* allele) were sampled. In these plants, the number of fertile florets in each spike was counted in the main shoot spikes at the UdL (where plants did not produce tiller spikes) and at both the main shoot and tiller spikes at the JIC. Plants were then separated into stems, leaves, and spikes, and their weights were determined after oven-drying at 65 °C for 72 h.

In order to illustrate the effects of treatments on floret development dynamics, we selected floret positions F1, F3, and F4 because the F1 floret normally develops completely to reach the stage of fertile florets in any condition and F3–F4 are more labile florets whose differences in developmental progress determine differences in spikelet fertility. The developmental patterns of the rest of the floret positions are shown in Supplementary Figs S3–S6 at *JXB* online.

### Statistical analysis

ANOVAs using JMP® Pro version 12.0 (SAS Institute Inc., Cary, NC, USA) were conducted to test differences between treatments. In addition, dynamics of leaf spikelet initiation and of leaf appearance, and the relationships between traits were analysed through regression analyses (depending on the particular cases, we used linear or bilinear regressions, in order to maintain a random distribution of residuals).

## Results

Growing the isogenic lines at temperatures of 6, 21, or 24 °C resulted in patterns of plant development with different types of abnormalities. Briefly, at 6 °C and 21 °C, abnormalities only became apparent during floret development, but only after the stage of the terminal spikelet (i.e. spikelet initiation proceeded normally at these temperatures). At 24 °C, abnormalities were evident at very early stages as none of the plants reached even the double ridge stage (for more details, see Ochagavía *et al.*, 2018*c*). Therefore, the quantitative analyses of the treatment effects on leaf and spikelet development were done with the whole range of temperatures explored except for 24 °C. As normal floret developmental was impaired at the three above-mentioned temperatures, the actions of, and interactions between, *Eps* alleles and temperatures on fertile florets and their determinants was limited to the range of thermal regimes which did not impair normal development until flowering (9 °C and 15 °C at the UdL, 12 °C and 18 °C at the JIC).

### Leaf appearance and time from flag leaf stage to heading

Final leaf number (FLN) was always low due to the conditions of the experiment: plants were vernalized and grown under long days. The difference in FLN between *Eps*-late and *Eps*-early NILs ranged from 0.11±0.25 to 0.75±0.26 leaves considering all temperature regimes where NILs reached flag leaf emergence. These extreme values corresponded to plants grown at 6 °C and 12 °C, respectively. Consequently, the difference in final leaf number between lines carrying *Eps-*late and *Eps-*early alleles did not explain the delay in time to heading due to the effect of the *Eps-*late allele.

**Fig. 2.**
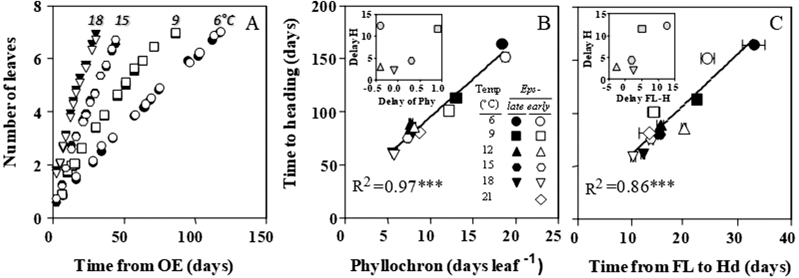
Dynamics of leaf appearance (for clarity this is restricted to the first seven leaves of plants grown under selected temperatures) from the onset of the experiment (OE) (A), and the relationship between time to heading and either average phyllochron (B) or time from the appearance of the flag leaf (FL) to heading (Hd) (C) for lines carrying *Eps-*early (open symbols) or *Eps*-late (filled symbols) alleles grown under constant temperatures of 6, 9, 12, 15, 18, and 21 °C. Lines in (B) in (C) were fitted by linear regression (*** means that the *R*^2^ value was highly significant, *P*<0.001). Inset in (B) and (C) is a detail of the delay produced by the *Eps-*late allele (difference between lines with the *Eps-*late or *Eps-*early alleles) in time to heading with respect to the delay in phyllochron (B) and in the duration of FL to Hd (C).

Therefore, the possible causes of the differences in time to heading are limited to effects of treatments on either phyllochron or the duration from the appearance of the flag leaf to heading. The dynamics of leaf appearance were strongly affected by growing temperature. In general, the leaves appeared very slowly (~0.05 leaves d^−1^) at 6 °C, and the rate of appearance increased (shifting the data points counter-clockwise in Fig. 2A) as the conditions became warmer until reaching a maximum rate (~0.20 leaves d^−1^) at 18 °C. However, leaves appeared at slower rates when grown at 21 °C and 24 °C (~0.15 and 0.12 leaves d^−1^, respectively). We did not find any consistent effect of *Eps* alleles on the rates of leaf appearance, and the very minor differences in the number of emerged leaves at particular times were inconsistent (Fig. 2A).

Time to heading was strongly related to both phyllochron (Fig. 2B) and time from the appearance of the flag leaf to heading (Fig. 2C). Once again, the major driver for these strong relationships was temperature through its universal effect on developmental rates, affecting all traits considered simultaneously and in the same direction, but only up to the optimum. The advancement of heading due to the action of *Eps*-early alleles, instead of *Eps-*late, could not be explained by its effect on phyllochron: the differences in phyllochron between NILs carrying *Eps-*late and *Eps*-early alleles were small and inconsistent (data points of NILs with *Eps-*late alleles were inconsistently on the right or the left of those corresponding to NILs with *Eps*-early alleles under the same temperature; Fig. 2B), as well as unrelated to the differences between the NILs in time to heading (*R*^2^=0.11, *P*=0.59; Fig. 2B, inset). On the other hand, when inspecting the relationship between time to heading and duration of the period from flag leaf emergence to heading, data points corresponding to NILs with *Eps-*late alleles were in general above and to the right of those corresponding to NILs with *Eps*-early alleles (Fig. 2C), with one exception at 12 °C. Thus, there was a positive relationship between the effect of the *Eps* alleles on time to heading and on the period between the appearance of the flag leaf and heading (*R*^2^=0.67, *P*=0.08; Fig. 2C, inset), suggesting in general that it is by changing the duration of this last part of the late reproductive phase that *Eps*-*D1* exerts its effect on ear emergence.

### Spikelet initiation and spikelets per spike

**Fig. 3.**
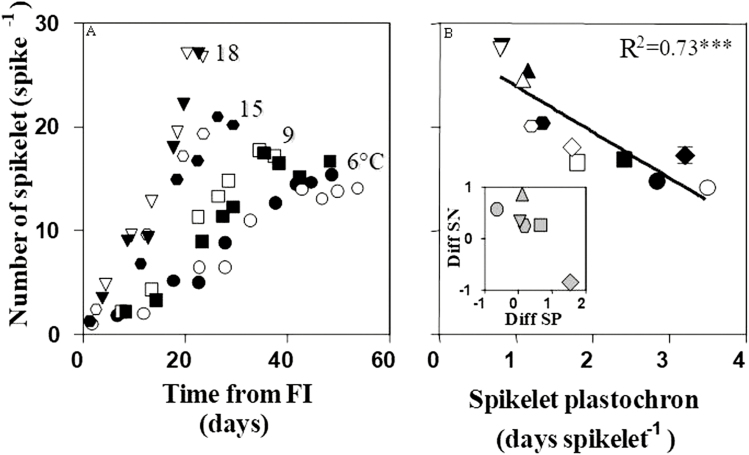
Dynamics of spikelet primordia initiation from floral initiation (FI) to terminal spikelet (for clarity this is restricted to selected temperatures) (A) and the relationship between the final number of spikelets per spike initiated and spikelet plastochron (B) for lines carrying *Eps*-early (open symbols) or *Eps-*late (filled symbols) alleles grown under constant temperatures of 6 (circles), 9 (squares), 12 (triangles), 15 (hexagon), 18 (inverted triangles), and 21 °C (diamond). The line in (B) was fitted by linear regression (*P*<0.001). The inset in (B) is a detail of the relationship between the differences (*Eps-*late minus *Eps*-early lines) in the number of spikelets (SN) per spike and in spikelet plastochron (SP).

Temperature treatments markedly affected the dynamics of spikelet initiation. The rate of spikelet primordia initiation was slowest at 6 °C (at ~0.3 primordia d^−1^); it increased with temperature until reaching a maximum rate at 18 °C (at ~1.2 primordia d^−1^) (Fig. 3A). Then spikelet primordia initiated at a slower rate at 21 °C (~0.4 primordia d^−1^).

Differences between *Eps-*late and *Eps*-early alleles in the rates of spikelet initiation were inconsistent, reflecting an *Eps*×temperature interaction. For instance, the dynamics at 18 °C were virtually the same for both alleles (Fig. 3A for the dynamics and Fig. 3B for differences in the abscissa for the average spikelet plastochron). At 15 °C and 12 °C, there was a trend for *Eps-*late lines to have slightly slower rates of spikelet initiation (i.e. a slightly longer spikelet plastochron) than their *Eps*-early counterparts (Fig. 3B). At the highest and second lowest temperatures explored, 21 °C and 9 °C, there was a clear difference between NILs in that *Eps-*late lines showed slower rates of spikelet initiation (i.e. a clearly longer spikelet plastochron) than *Eps*-early lines (Fig. 3B). However, beyond these quantitative interactions in spikelet initiation rates, the inclusion of a rather low temperature revealed a qualitative interaction as well: at 6 °C, the effect of *Eps* alleles on the rate of spikelet initiation was reversed; the NILs carrying *Eps-*late alleles showed faster rates of spikelet initiation (i.e. clearly shorter spikelet plastochron) than those with *Eps*-early alleles (Fig. 3B).

The final outcome of the number of spikelets per spike is a consequence of the balance of the effects of treatments on its two components: the spikelet plastochron and the duration of the early reproductive phase from floral initiation to terminal spikelet. As temperature affects both, they were strongly and positively correlated (Supplementary Fig. S1). For treatments to have affected the number of spikelets per spike, these components should differ in their sensitivity. Comparing the range of variation in both components, it seems evident that the duration of spikelet plastochron was more responsive than the duration of the phase of spikelet initiation (the former varied from less than ~0.8 d to ~3.5 d per spikelet, i.e. >400%, whilst the latter varied from ~23 d to ~57 d, i.e. <250%). The number of spikelets per spike was strongly related to both components (Fig. 3B; Supplementary Fig. S2); again these relationships were mainly driven by temperature, producing, in both cases, negative trends. This confirms that the effect of temperature on the duration of spikelet plastochron was more relevant than that on the duration of the early reproductive phase (otherwise the latter relationship should have been positive rather than negative). Differences between *Eps*-early and *Eps-*late alleles in spikelets per spike were relatively minor (Fig. 3). Within these minor differences it seemed that differences in spikelet plastochron (*r*= –0.84, *P*<0.05, Fig. 3B, inset) and in duration of the early reproductive phase (*r*= –0.82, P<0.05, Supplementary Fig. S2, inset) similarly explained the minor effect of this *Eps* gene in spikelets per spike, only considering the coefficients of determination. However, in physiological terms, differences between NILs carrying *Eps*-early and *Eps-*late alleles on spikelet plastochron were the relevant ones. That is, the reason why the contrasting NILs differed in their number of spikelets per spike was their difference in spikelet plastochron; NILs with a longer plastochron ended up having fewer spikelets. On the other hand, the negative relationship between spikelets per spike and the duration of the phase of spikelet initiation is counter-intuitive and simply reflects that the dominant component determining the slight effect on spikelets per spike was that on spikelet plastochron. As the outcome of the effects of this *Eps* gene, it produced a sort of trade-off and then changes produced in spikelets per spike were relatively marginal and the effects on floret development were critical for the ultimate effect of this gene on spike fertility (see below).

### Fertile florets at anthesis

**Fig. 4.**
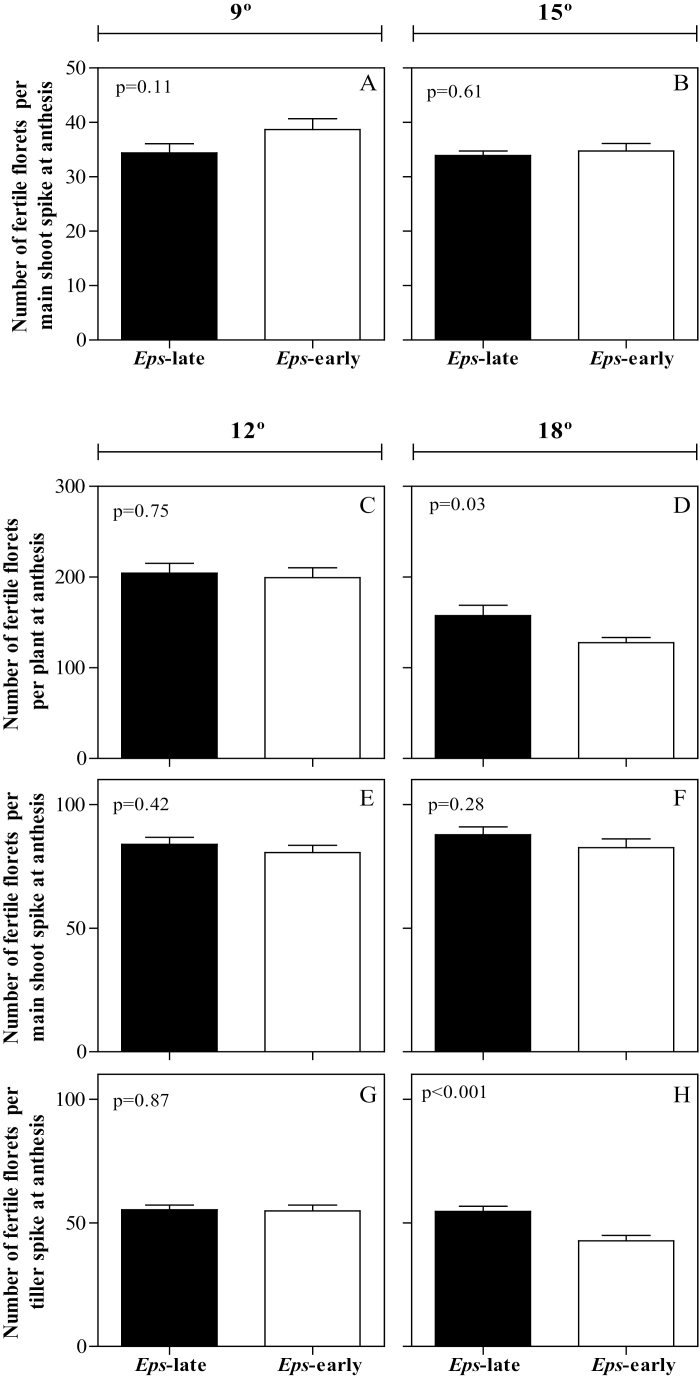
Number of fertile florets per plant at anthesis [only the main shoot spike in the UdL (A, B) at 9 °C (A) and 15 °C (B), and total (C, D) combining main shoot (E, F) and tiller spikes (G, H) in the JIC at 12 °C (C, E, G) and 18 °C (D, F, H)] between the *Eps* NILs carrying either the *Eps*-late (filled bars) or *Eps*-early alleles (open bars). Error bars stand for the SE of the means. *P*-values resulting from a *t*-test are shown inside each panel.

There was a detectable *Eps*×temperature interaction for the number of fertile florets at anthesis in each of the two locations. At 9 °C in the UdL, there was a trend (the difference was significant only at ~10% probability threshold) for NILs with *Eps*-early alleles to have more fertile florets per spike than NILs with *Eps*-late alleles (Fig. 4A). On the other hand, there was no difference in the number of fertile florets between NILs at 15 °C (Fig. 4B). In the JIC, the number of fertile florets per plant was virtually the same for both NILs when growing at 12 °C (Fig. 4C, E, G). However, NILs differed significantly in fertile florets per plant when growing at 18 °C (Fig. 4D), with larger effects observed on primary tillers (Fig. 4H) than on main shoot spikes (Fig. 4F). However, rather relevantly, the nature of the interactions in both locations was opposite: while at 18 °C the NILs with the *Eps*-late alleles tended to have more fertile florets than those with the *Eps*-early alleles, at 9 °C the *Eps*-late alleles decreased the number of fertile florets (cf. Fig. 4A and D).

**Fig. 5.**
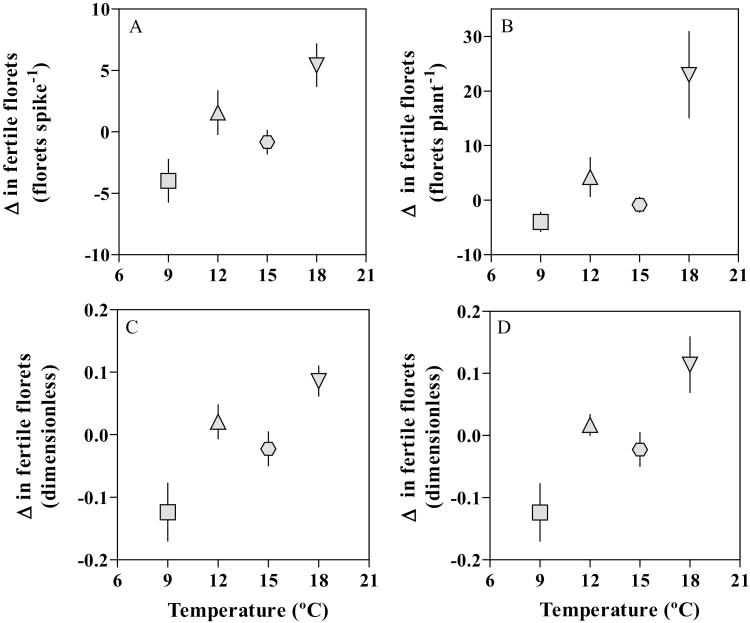
Differences in the number of fertile florets per spike (A, C) and per plant (B, D) at anthesis between the NILs carrying the *Eps*-late and the *Eps*-early alleles plotted against growing temperature, in both absolute (A, B) and relative values (C, D). Each data point is the average of all plants of all replicates (18), and the segment in each data point stands for the SE of the means.

Indeed, if we consider the four temperatures together, more clear evidence of the temperature effect on the impact of this *Eps* gene on the final outcome for floret development emerges. We have done so by calculating the difference in number of fertile florets between NILs carrying the late *Eps* allele and those carrying the early allele, in both absolute and relative terms, with the growing temperature (Fig. 5). The impact of temperature on the effect of the *Eps* alleles on the number of fertile florets was noticeable in quantitative and qualitative terms. Quantitatively, there was a clear positive trend, largely linear, of the difference with the growing temperatures (i.e. the higher the temperature the larger the difference in favour of the NILs with the *Eps*-late alleles; Fig. 5). The qualitative impact of temperature on the effects of *Eps* alleles on fertile florets is shown by the fact that the relationship explored both positive and negative values of the difference in fertile florets (Fig. 5); when negative—at the lowest temperature analysed—it implies that, compared with the NILs carrying the *Eps*-early alleles, the *Eps*-late alleles reduced the number of fertile florets, and when positive—at the highest temperature analysed—it reflects that these alleles increased the number of fertile florets per spike and per plant. At the intermediate temperatures, the differences were negligible (Fig. 5).

### ‘Mapping’ fertile florets

**Fig. 6.**
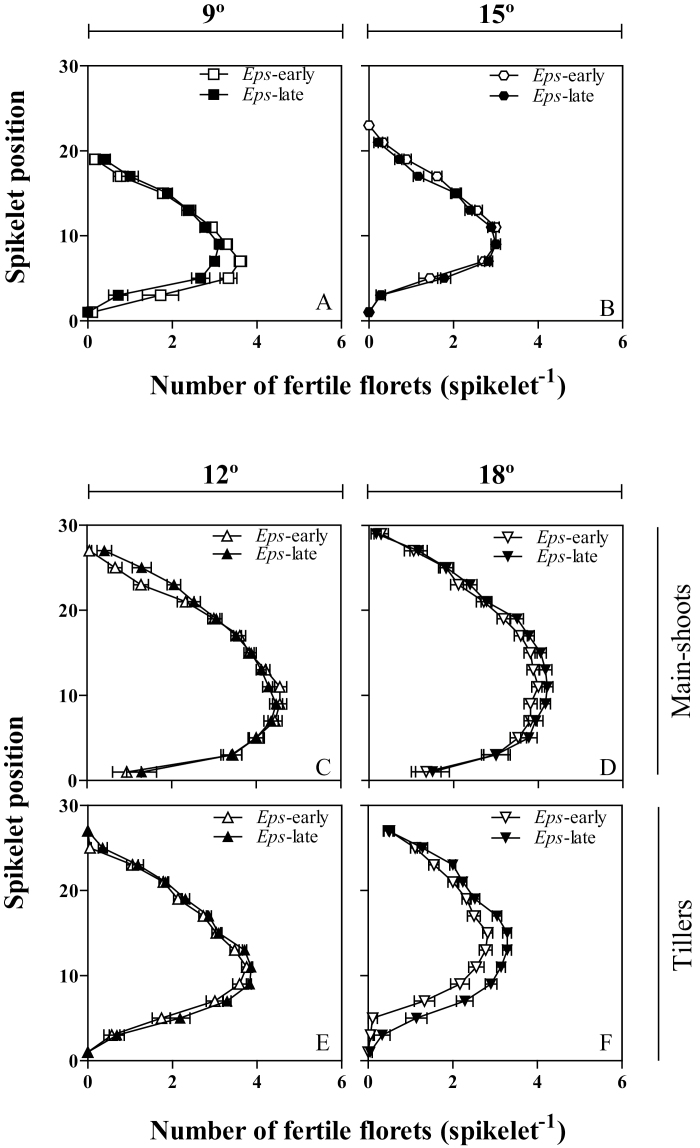
Number of fertile florets in each spikelet position on the main shoot spike in the UdL (A, B) at 9 °C (A) and 15 °C (B), and on the main shoot (C, D) and tiller spikes (E, F) in the JIC at 12 °C (C, E) and 18 °C (D, F) between the *Eps* NILs carrying either the *Eps*-late (filled symbols) or *Eps*-early alleles (open symbols). Each data point is the average of all the plants of all the replicates (18), and the segment in each data point stands for the SE of the means.

In the experiments carried out at the UdL, the trend to increase the number of fertile florets per spike due to the action of the *Eps*-early alleles at the lowest temperature (9 °C) was clear in the bottom half of the spike, in which the difference was significant in a number of spikelets (Fig. 6A). At 15 °C, where the differences in florets per spike were negligible, there was no clear differences between NILs with *Eps*-late and *Eps*-early alleles at any of the spikelets (Fig. 6B).

Regarding the experiments conducted at the JIC, at 12 °C all NILs had a very similar number of fertile florets per spikelet (Fig. 6C, E), while at the warmer regime (18 °C) the *Eps*-late NILs showed more fertile florets per spike than those with *Eps*-early alleles, particularly in the tiller spikes (Fig. 6D, F).

### Developmental dynamics of individual floret primordia and of living florets

**Fig. 7.**
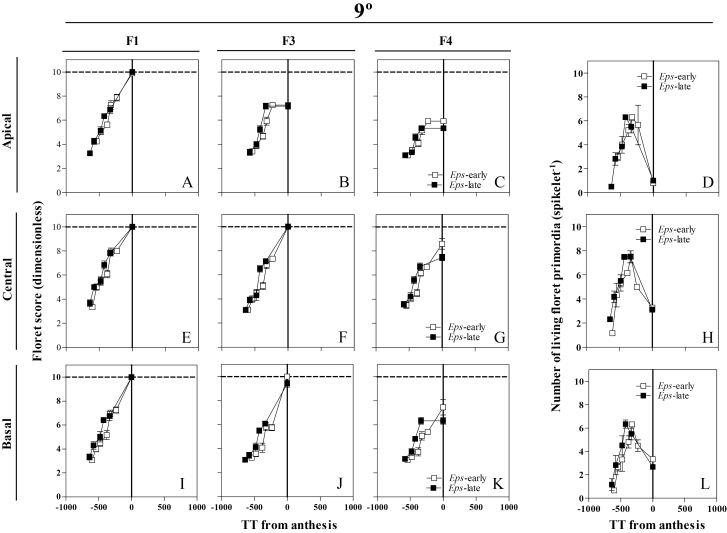
Dynamics of the floret development of florets F1, F3, and F4 (A–C, E–G, I–K) and the number of living floret primordia (D, H, L) through thermal time from anthesis in the apical (A–D), central (E–H), and basal (I–L) spikelets between NILs carrying either the *Eps*-late (filled circles) or early variant (open triangles) growing at 9 °C. Each data point is the average of two plants per three replicates, and the segment in each data point stands for the SE of the means.

**Fig. 8.**
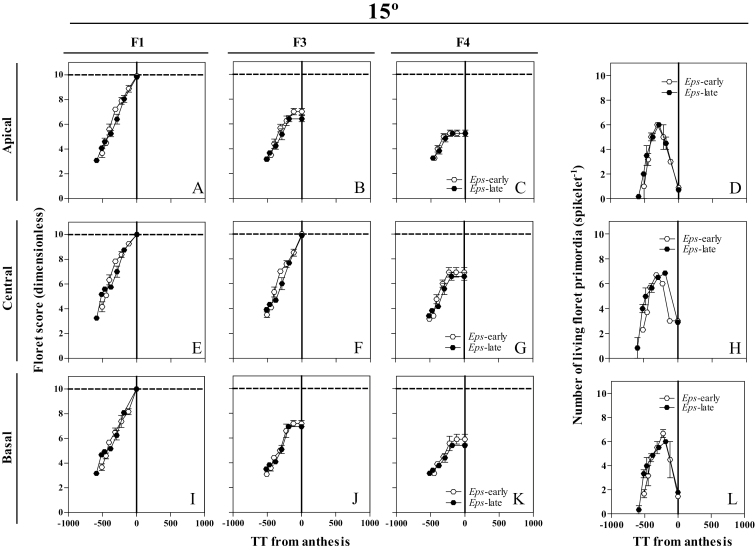
Dynamics of the floret development of florets F1, F3, and F4 (A–C, E–G, I–K) and the number of living floret primordia (D, H, L) through thermal time from anthesis in the apical (A–D), central (E–H), and basal (I–L) spikelets between NILs carrying either the *Eps*-late (filled circles) or early variant (open triangles) growing at 15 °C. Each data point is the average of two plants per three replicates, and the segment in each data point stands for the SE of the means.

At 9 °C, floret development dynamics between NILs of the different florets (F1, F3, and F4) and from different spikelet positions (apical, central, and basal) showed slight but noticeable differences (Fig. 7A–C, E–G, I–K; Supplementary Fig. S3): F1 in the apical spikelets which was fertile in all the plants with the *Eps*-late allele and only in 78% of those with the *Eps*-early allele (Fig. 7A); and F3 in the basal position which was always fertile in the *Eps*-early NILs but only in 67% of the *Eps*-late NILs (Fig. 7J). In addition, the *Eps*-early lines tended to present advanced stages of floret development in F4 in central spikelets; 25% of the plants reached the fertile stage compared with just 8% with *Eps*-late alleles; in the basal positions, 33% of the plants with *Eps*-early alleles reached the fertile stage while none with *Eps*-late alleles reached that stage (Fig. 7G, K). Moreover, the length of the floret development phase, the maximum number of floret primordia developed, and the final number at anthesis differed between NILs for the apical and central spikelets (Fig. 7A–H), but in the basal spikelets the *Eps*-early NILs produced a higher number of fertile florets at anthesis than the *Eps*-late NILs (Fig. 7I–L). On the other hand, at 15 °C, there were no differences in floret development between NILs carrying the *Eps*-early and -late alleles (Fig. 8A–C, E–G, I–K; Supplementary Fig. S4) or in the number of living floret primordia at 15 °C (Fig. 8D, H, L) and the length of the floret development phase or in the maximum or final floret number.

**Fig. 9.**
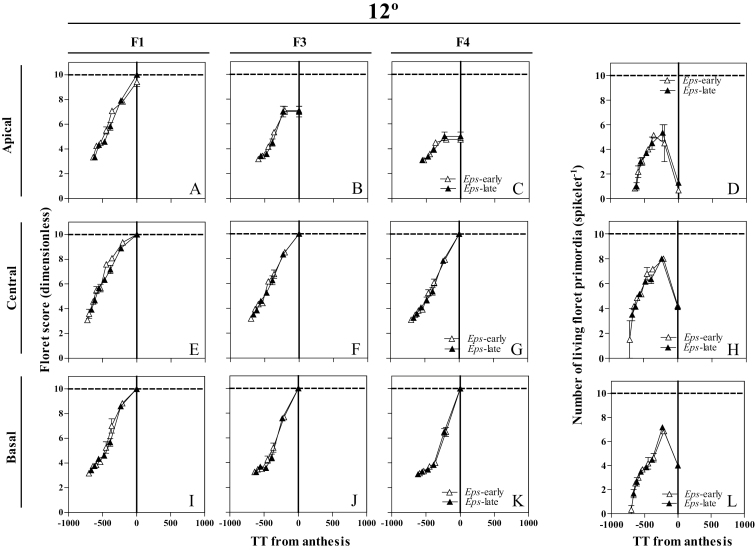
Dynamics of the floret development of florets F1, F3, and F4 (A–C, E–G, I–K) and the number of living floret primordia (D, H, L) through thermal time from anthesis in the apical (A–D), central (E–H), and basal (I–L) spikelets between NILs carrying either the *Eps*-late (filled circles) or early variant (open triangles) growing at 12 °C. Each data point is the average of two plants per three replicates, and the segment in each data point stands for the SE of the means.

**Fig. 10.**
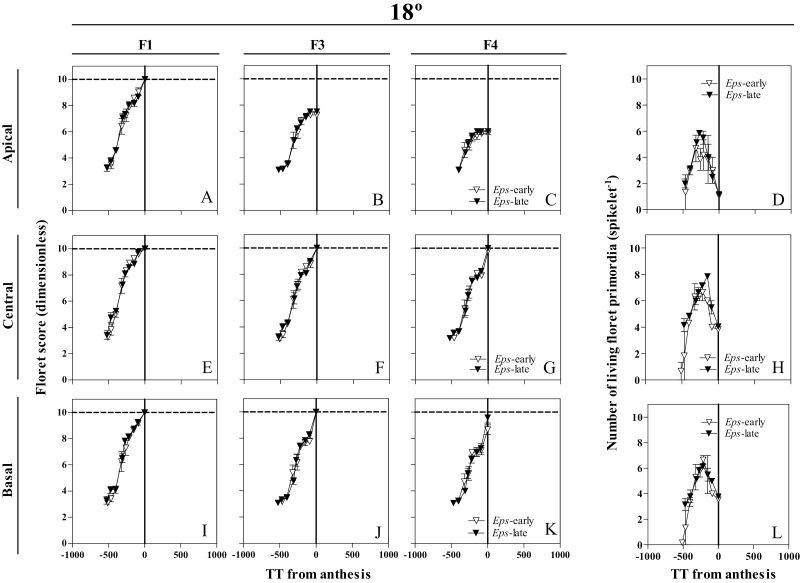
Dynamics of the floret development of florets F1, F3, and F4 (A–C, E–G, I–K) and the number of living floret primordia (D, H, L) through thermal time from anthesis in the apical (A–D), central (E–H), and basal (I–L) spikelets between NILs carrying either the *Eps*-late (filled circles) or early variant (open triangles) growing at 18 °C. Each data point is the average of two plants per three replicates, and the segment in each data point stands for the SE of the means.

At 12 °C, only very slight differences were observed in F1 and F2 of the apical spikelets. F1 developed normally to reach the stage of fertile floret in all NILs with the *Eps*-late alleles, while in the *Eps*-early NILs it developed normally to reach that stage in most of the plants (Fig. 9A). F2 developed normally to become a fertile floret in ~25% of the plants of the NILs with the *Eps*-late alleles, but in none of the plants of *Eps*-early NILs (Fig. S5A), which was reflected in a slight difference in final fertile floret number in apical spikelets (Fig. 9D). In central and basal spikelets, no clear differences were observed in floret development between NILs with different *Eps* alleles (Fig. 9; Supplementary Fig. S5). At 18 °C, the main differences between NILs carrying either the *Eps*-late or -early alleles were in the central spikelets where F4 of all the plants of NILs carrying the *Eps*-late alleles developed normally until reaching the fertile floret stage (and even 8% of the plants of these NILs presented a F5 that developed normally to reach the fertile floret stage; Supplementary Fig. S6) while only 85% of the *Eps*-early NILs showed F4 developing normally until reaching the fertile floret stage (Fig. 10C, G, K). In addition, in basal spikelets, F4 florets developed to fertility in 83% of the plants of *Eps*-late NILs, while this figure was just 53% for the *Eps*-early NILs (Fig. 10K). Consequently, at this temperature, the NILs carrying the *Eps*-late allele developed a higher maximum number of floret primordia than those with the *Eps*-early allele in the apical and central positions (Fig. 10A–H) and tended to present a higher final number of fertile florets in all the positions (Fig. 10D, H, L).

### Number of fertile florets and spike dry weight at anthesis

**Fig. 11.**
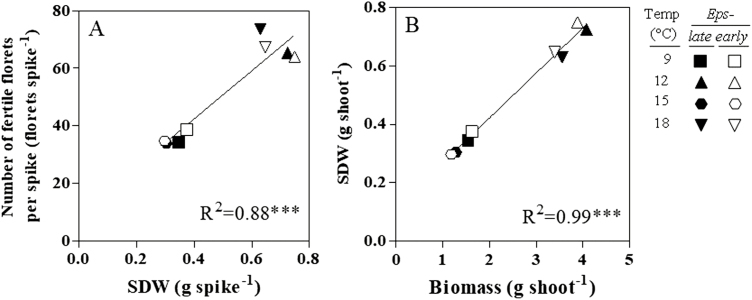
Relationship between the number of fertile florets per spike and the spike dry weight at anthesis (SDW, A) and relationship between the SDW and the biomass at anthesis (B) for the NILs carrying either the *Eps*-late or *Eps*-early alleles growing at 9, 12, 15, and 18 ° C. Each data point is the average of all the plants of all replicates left for the final determinations at anthesis (18). Lines were fitted by linear regression, and the corresponding equations, coefficient of determination (*R*^2^), and the level of significance (*P*-value) are shown.

A strong positive relationship was found between the number of fertile florets and the spike dry weight at anthesis (SDW) (Fig. 11A), which in turn were related to differences in growth more than in partitioning (Fig. 11B).

However, many of the relationships were driven by the differential growth between the growth chambers in the JIC (right-top data points) and the UdL (left-bottom data points); and, within each of the specific chambers used, the relationship between spike dry weight and total biomass at anthesis was also driven by temperature (a lower temperature produced heavier plants with heavier spikes; Fig. 11B). The *Eps* alleles did not affect partitioning to the reproductive organs and therefore differences in SDW between NILs with different *Eps* alleles were negligible within each temperature condition (Fig. 11). On the other hand, within each of the growth chambers, the differences in spike fertility, when detected (at 9 °C, cf. ‘a’ and ‘b’; and at 18 °C, cf. ‘g’ and ‘h’; Fig. 11A), were not due to allelic effects on spike dry weight (Fig. 11A). In fact in the chamber at 9 °C (where the *Eps*-early alleles increased the number of fertile florets) the early lines produced an average of 103.6 fertile florets per gram of spike (g_spike_^−1^) at anthesis, slightly higher than the 100.9 florets g_spike_^−1^ of the late lines; whilst in the chamber at 18 °C (in which the difference in favour of the *Eps*-late lines was maximized) the late lines produced a higher average number of fertile florets per gram of spike at anthesis than the early lines (123.8 and 113.1 florets g_spike_^−1^, respectively).

## Discussion

An earliness *per se* gene located on chromosome 1DL (*Eps-D1*) has been shown to be useful for fine-tuning wheat adaptation to particular locations/conditions by producing relatively minor changes in time to anthesis (Griffiths *et al.*, 2009; Zikhali *et al.*, 2014; Ochagavía *et al.*, 2018*a*). It is well understood that temperature strongly affects the rate of growth and development in cereals (Slafer and Rawson, 1995; Porter and Gawith, 1999; Porter and Semenov, 2005; Kiss *et al.*, 2017), including effects on spike fertility (Fischer, 1985; Tashiro and Wardlaw, 1990; Ugarte *et al.*, 2007; Prasad and Djanaguiraman, 2014). However, less is known about how temperature can modulate the action of specific alleles on developmental traits, such as the effects of *Eps-D1* on time to heading and its component phases (Ochagavía *et al.*, 2018*c*, and references therein). Even less is known about the effects of *Eps* genes on the initiation of vegetative organs (Ochagavía *et al.*, 2018*a*, and references therein) and on spikelet fertility (Prieto et al., 2018*b*, and references therein). This information is important for the design of strategic crosses aimed at adapting genotypes to a target environment. Therefore, knowing the effect of particular adaptation genes on leaf number and phyllochron with ramifications for grain yield determinants such as spike fertility may facilitate the selection of the best available alleles for incorporation into new varieties with improved adaptation and yield potential (or at least to improve adaptation whilst minimizing losses in yield potential). Understanding the effect of the *Eps* genes×temperature interaction on dynamics of leaf initiation and appearance and on setting levels of spike fertility is a complex challenge. This is mainly because the additive effects of these genes are unsurprisingly small (Ochagavía *et al.*, 2018*a*; Prieto *et al.*, 2018*b*).

The parallelism between the effects in total time to heading and in the late reproductive phase (Ochagavía *et al.*, 2018*a*) is commensurate with the finding that this *Eps* gene affected more consistently the period from flag leaf emergence to heading than phyllochron, reinforcing the point that the effect on the duration of the late reproductive phase may well be concentrated in the last part of the stem elongation period, a phase that might be particularly influential in determining spike fertility and grain number (Kirby, 1988).

Moreover, in the present study, the magnitude of the effects of *Eps* alleles on spike fertility seemed to be qualitative: depending on the growing temperature, the particular *Eps* alleles were shown to increase, decrease, or have no effect at all on the number of spikelets or florets and the consequent integration of these processes on spike fertility. Regarding the effects of *Eps* alleles across temperatures on spikelet number per spike, even though there tended to be a trade-off between its effects on spikelet plastochron and duration of early reproductive phase, those on spikelet plastochron were stronger, generating narrow differences in the number of spikelets per spike between alleles. The finding of a compensation between the effects of *Eps* genes on spikelet plastochron and on duration of the early reproductive phase had been also found in the field, though under a single temperature regime (Ochagavía *et al.*, 2018*a*), and is also in line with the effects of *Ppd-1* genes (e.g. Ochagavía *et al.*, 2018*b*; Pérez-Gianmarco *et al.*, 2018). Lewis *et al.* (2008) associated an increased number of spikelets per spike by the *Eps-A*^*m*^*1*-late allele from diploid wheat with an increased duration of the spike development phase, but also spikelet plastochron (as the impact on spikelets per spike was much smaller than that on the duration from the double ridge and terminal spikelet).

Based on the results of this work which identified strong *Eps*×temperature interaction, the final effects of *Eps-D1* on fertile floret number was complex and their use in combining fine-tuned genotypes in terms of adaptation together with maximum spike fertility would be highly dependent upon growing temperature. At the lowest temperature tested, the *Eps*-early alleles had more fertile florets than those with the *Eps*-late alleles, whilst at the highest temperature, at which floret development progressed more rapidly, the *Eps*-late alleles increased the number of fertile florets (and at intermediate temperatures there was no effect on spike fertility). In barley, Ejaz and von Korff (2017) found that early lines accelerated flowering time but maintained the number of fertile florets under high temperatures (28/24 °C).

We showed that, regardless of the interaction with temperature, the effects of this *Eps* gene on the number of fertile florets was more closely associated with floret survival mechanisms. So the allele that increased fertile floret number also increased the likelihood that labile floret primordia would continue their development normally. On the other hand, there was no consistent effect on the number of primordia initiated. This is in agreement with most of the literature, which shows that the most critical step determining spike fertility is the capacity to sustain normal development in labile florets. For instance, spike fertility increased due to improved survival of floret primordia, with no effects on the maximum number of florets initiated in response to increased spike growth due to either genotypic (e.g. introgression of *Rht* genes increasing dry matter partitioning to the juvenile spikes; Fischer and Stockman, 1980; Siddique *et al.*, 1989; Slafer and Andrade, 1993; Miralles *et al.*, 1998) or environmental factors (e.g. due to fertilizing with N or reducing competition with detillering plants increasing shoot biomass and not affecting partitioning; Ferrante et al., 2010, 2013*a*, *b*). Furthermore, treatments modifying wheat development during floret development (such as modifying daylength during stem elongation; e.g. Miralles *et al.*, 2000; González et al., 2003, 2005) or variation among elite germplasm in duration of this phase when floret development takes place (e.g. González-Navarro *et al.*, 2015; Guo *et al.*, 2016) also affected spike fertility by changing floret survival more than floret initiation. In addition, the introgression of genes affecting developmental responses to environment also seemed to affect spike fertility more through changes in floret survival than floret initiation (e.g. González *et al.*, 2011; Prieto et al., 2018*a* when considering Ppd alleles and Prieto et al., 2018*b* with *Eps* alleles under a single temperature in the field). The reason why floret survival dominates initiation in terms of explaining the effects of genetic and environmental factors on spike fertility has evolutionary roots (see discussion in Sadras and Slafer, 2012).

The influence of these *Eps* alleles on the survival of fertile florets seemed to be largely independent of changes in pre-anthesis spike growth. Unlike the cases mentioned above, in which changes in growth and/or development were mostly quite large, the effects of *Eps* genes are much more subtle. In this case, the effect on survival of labile floret primordia was related to the efficiency with which spike dry weight was used to set a particular level of spike fertility. This is commensurate with the differences in spike fertility between elite lines of wheat, where differences are also much more subtle compared with studies comparing lines within diversity panels and wide crosses, in which fruiting efficiency (Slafer *et al.*, 2015) seemed much more relevant than spike dry matter to explain genetic differences in spike fertility (Elía *et al.*, 2016). A hypothetical framework for these observations could be that when the *Eps* genes increase the rate of development of individual florets (and, depending on the temperature, whether that function would correspond to the early or late allele) it allows a few labile florets (which, if developing at a slower rate would die) to continue developing normally and eventually become fertile florets, therefore improving the efficiency of spike dry weight to reach a higher level of fertility.

An empirical consequence of this analysis is that breeders would benefit considerably by conducting a better characterization of many of the *Eps* genes not only for their impact on time to heading and on dynamics of leaf appearance (both critical for fine-tuning adaptation) but also for their effects on the developmental rates of processes determining *a posteriori* yield components as well as for their interactions with temperature (as, knowing the expected temperature during stem elongation in the target populations of environments, they would be able to predict the impact of these genes on spike fertility, beyond their effects on time to heading). This knowledge would allow breeders to optimize strategic crosses by maximizing the likelihood of identifying in the progeny lines with a better combination between adaptation and simultaneously increased spike fertility.

## Supplementary data

Supplementary data are available at *JXB* online.

Fig. S1. Relationship between spikelet plastochron and the duration of the early reproductive phase.

Fig. S2. Relationship between the final number of spikelets per spike initiated and duration of the early reproductive phase from FI to the terminal spikelet.

Fig. S3. Dynamics of the floret development of florets F2, F5, F6, F7, and F8 at 9 °C.

Fig. S4. Dynamics of the floret development of floret F2, F5, F6, F7, and F8 at 15 °C.

Fig. S5. Dynamics of the floret development of florets F2, F5, F6, F7, and F8 at 12 °C.

Fig. S6. Dynamics of the floret development of florets F2, F5, F6, F7, and F8 at 18 °C.

erz568_suppl_Supplementary_Figures_Tables

## Abbreviations:

Eps: earliness *per se*
FLN: final leaf number
JIC: John Innes Centre
NIL: near isogenic line
Ppd: photoperiod
SDW: spike dry weight at anthesis
UdL: University of Lleida
Vrn: vernalization
