# Supplementary material for: Earliness per se×temperature interaction: consequences on leaf, spikelet, and floret development in wheat
Source: J Exp Bot. 2019 Dec 26;71(6):1956–68. doi: 10.1093/jxb/erz568 (PMC7242086; doi:10.1093/jxb/erz568)
Supplement: erz568_suppl_Supplementary_Figures_Tables [file erz568_suppl_supplementary_figures_tables.pdf]

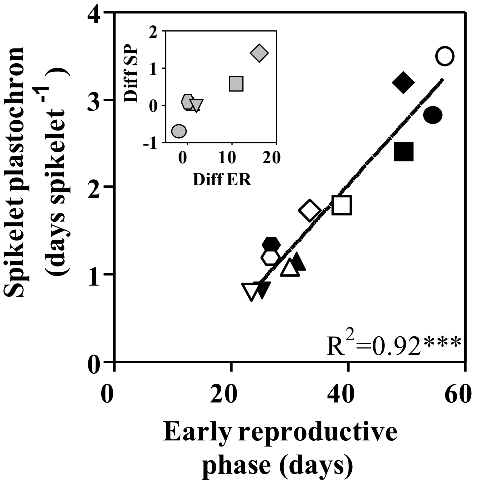

Fig. S1. Relationship between spikelet plastochron and the duration of the early reproductive phase for lines carrying *Eps*-early (open symbols) or *Eps*-late (closed symbols) alleles grown under constant temperatures of 6 (circles), 9 (squares), 12 (triangles), 15 (hexagon), 18 (inverted triangles) and 21°C (diamond). Line fitted by linear regression (*P<0. 001*). Inset is a detail of the relationship between the differences (*Eps*-late minus *Eps*-early lines) in spikelet plastochron (SP) and in duration of the early reproductive phase (ER).

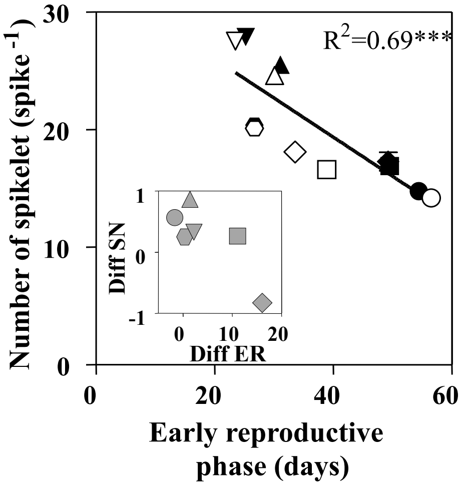

Fig. S2. Relationship between the final number of spikelets per spike initiated and duration of the early reproductive phase from FI to terminal spikelet for lines carrying *Eps*-early (open symbols) or *Eps*-late (closed symbols) alleles grown under constant temperatures of 6 (circles), 9 (squares), 12 (triangles), 15 (hexagon), 18 (inverted triangles) and 21°C (diamond). Line was fitted by linear regression ($P<0.001$). Inset is a detail of the relationship between the differences (*Eps*-late minus *Eps*-early lines) in number of spikelets (SN) per spike and in duration of the early reproductive phase (ER).

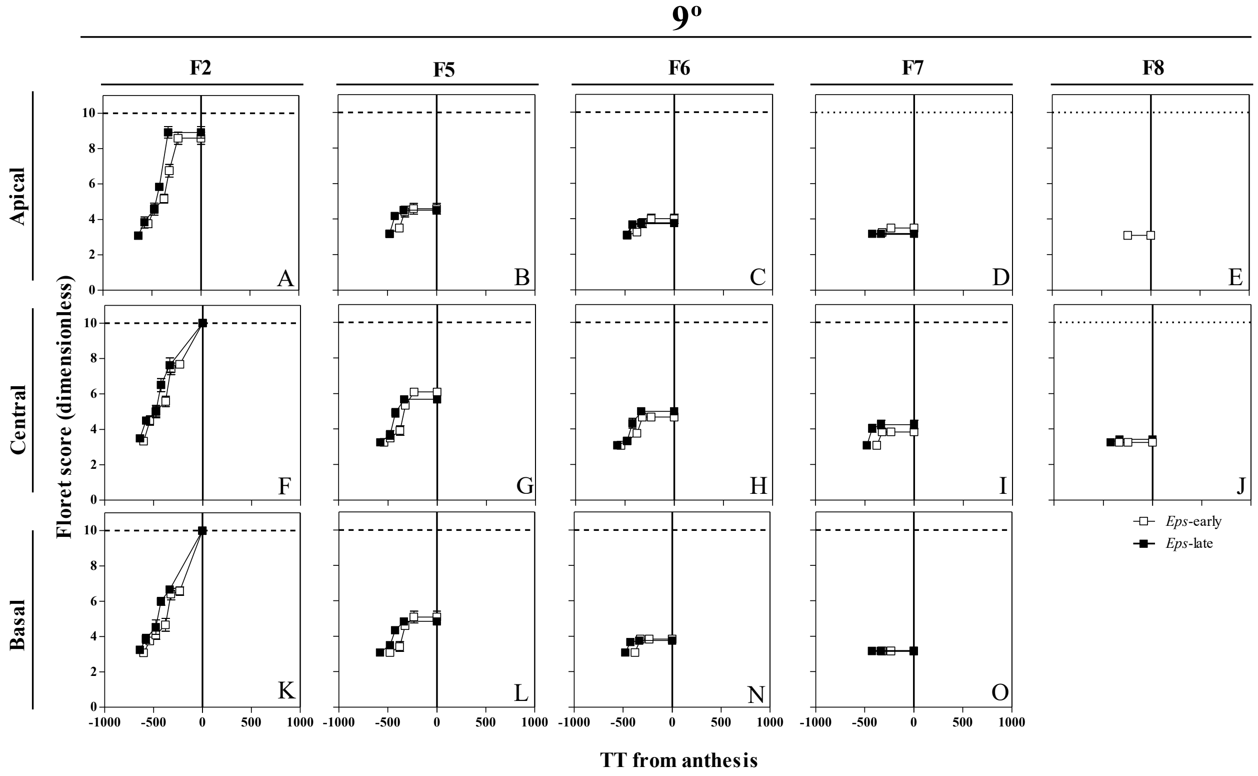

Fig. S3. Dynamics of the floret development of floret F2, F5, F6, F7 and F8 through thermal time from anthesis in the apical (A, B, C, D, E), central (F, G, H, I, J) and basal (K, L, N, O) spikelets between NILs carrying either the *Eps*-late (closed circles) or early variant (open triangles) growing at 9º. Each data-point is the average of 2 plants per 3 replicates and the segment in each data-point stands for the standard error of the means.

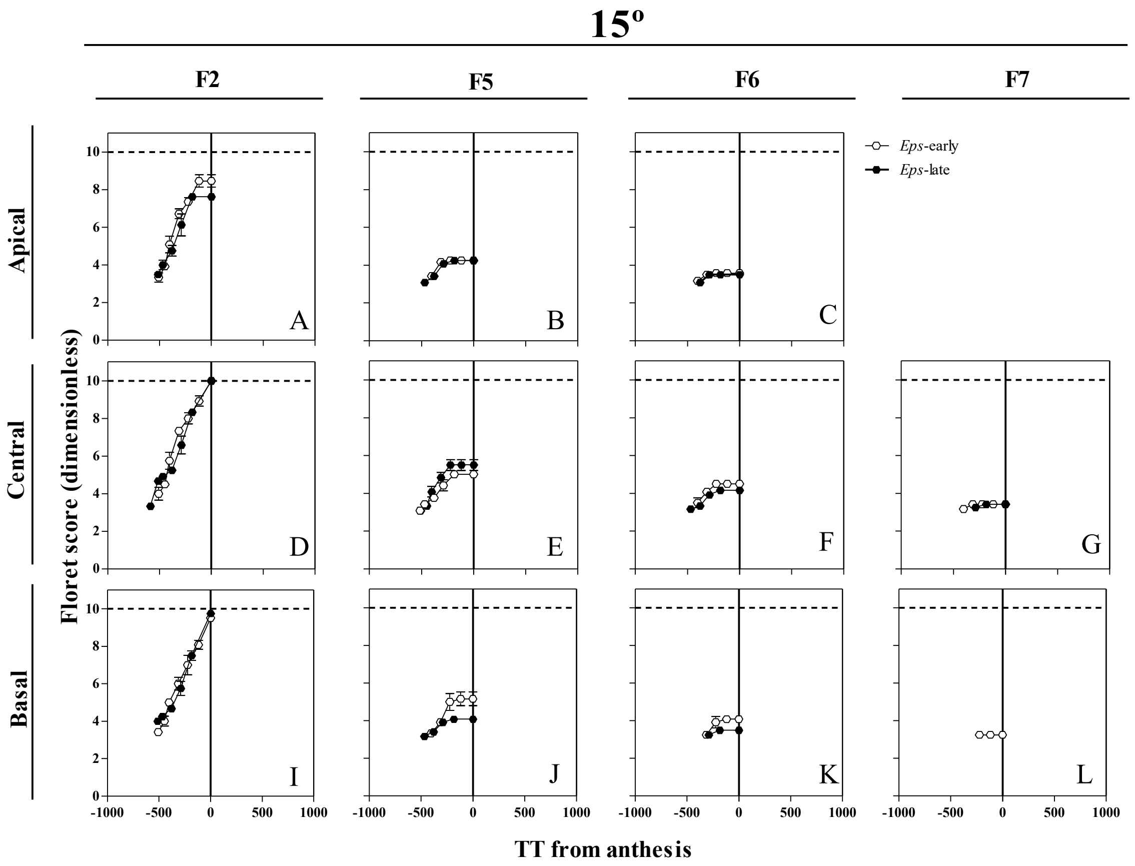

Fig. S4. Dynamics of the floret development of floret F2, F5, F6 and F7 through thermal time from anthesis in the apical (A, B, C), central (D, E, F, G) and basal (I, J, K, L) spikelets between NILs carrying either the *Eps*-late (closed circles) or early variant (open triangles) growing at 15º. Each data-point is the average of 3 plants per replicate, bars stands for the standard error of the means. Each data-point is the average of 2 plants per 3 replicates and the segment in each data-point stands for the standard error of the means.

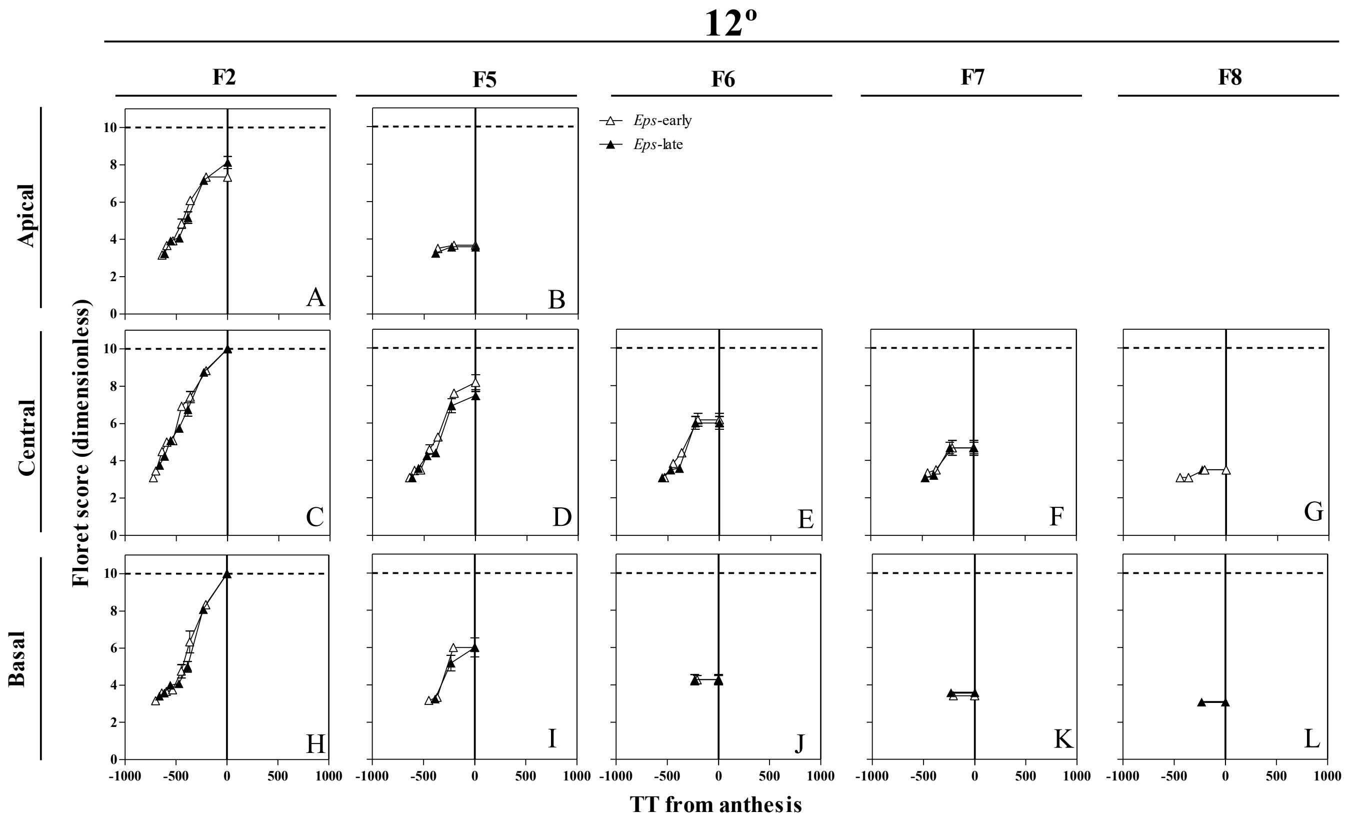

Fig. S5. Dynamics of the floret development of floret F2, F5, F6, F7 and F8 through thermal time from anthesis in the apical (A, B), central (C, D, E, F, G) and basal (H, I, J, K, L) spikelets between NILs carrying either the *Eps*-late (closed circles) or early variant (open triangles) growing at 12º. Each data-point is the average of 2 plants per 3 replicates and the segment in each data-point stands for the standard error of the means.

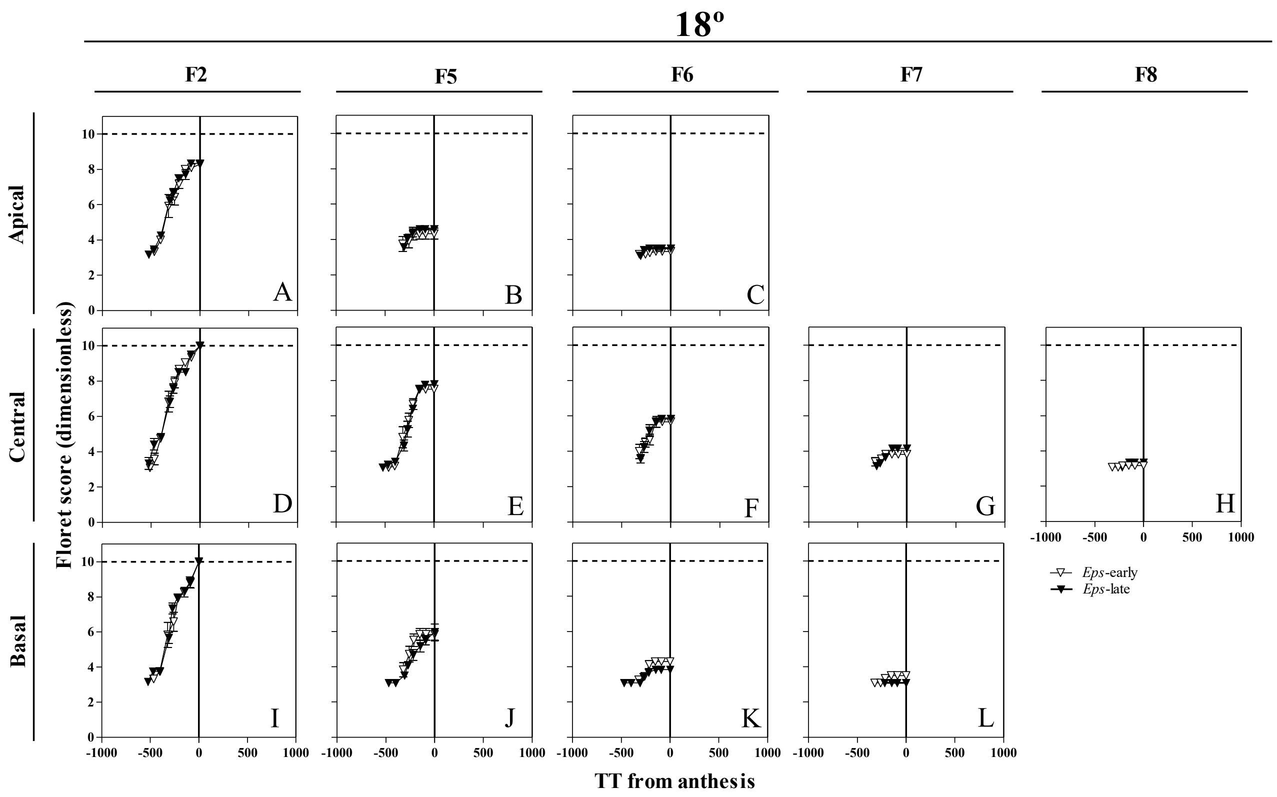

Fig. S6. Dynamics of the floret development of floret F2, F5, F6, F7 and F8 through thermal time from anthesis in the apical (A, B, C), central (D, E, F, G, H) and basal (I, J, K, L) spikelets between NILs carrying either the *Eps*-late (closed circles) or early variant (open triangles) growing at 18º. Each data-point is the average of 2 plants per 3 replicates and the segment in each data-point stands for the standard error of the means.
